# Atypical methotrexate ulcerative stomatitis with features of lymphoproliferative like disorder: Report of a rare ciprofloxacin-induced case and review of the literature

**DOI:** 10.4317/jced.52909

**Published:** 2016-12-01

**Authors:** Nikolaos Katsoulas, Evanthia Chrysomali, Evangelia Piperi, Georgia Levidou, Alexandra Sklavounou-Andrikopoulou

**Affiliations:** 1 DDS, MSc, MSc in Oral Medicine and Pathology, Department of Oral Medicine and Oral Pathology, School of Dentistry, National and Kapodistrian University of Athens, Athens, Greece; 2 DDS, PhD, Assistant Professor, Department of Oral Medicine and Oral Pathology, School of Dentistry, National and Kapodistrian University of Athens, Athens, Greece; 3 DDS, MSc, PhD, Assistant Professor, Department of Oral Medicine and Oral Pathology, School of Dentistry, National and Kapodistrian University of Athens, Athens, Greece; 4 MD, PhD, Consultant Hematopathologist, 1st Department of Pathology, School of Medicine, National and Kapodistrian University of Athens, Athens, Greece; 5 DDS, MSc, PhD, Professor, Department of Oral Medicine and Oral Pathology, School of Dentistry, National and Kapodistrian University of Athens, Athens, Greece

## Abstract

Methotrexate (MTX) is an established immunomodulating agent used in low doses (LDMTX) to treat several autoimmune diseases. Ulcerative stomatitis (US) may be observed as a long-term LDMTX adverse effect showing a wide histopathologic spectrum. A 73-year old female presented with painful oral ulcers of 5 days duration. The patient had been under treatment for rheumatoid arthritis with LDMTX, while one week before presentation she was prescribed ciprofloxacin for a urinary infection. Histopathologic examination of a lingual ulcer revealed a polymorphous lymphohistiocytic proliferation with scattered binucleated atypical lymphocytes. Immunohistochemically, most cells were of T-cell lineage while the EBER test was negative and a diagnosis of MTX-induced reactive ulceration was rendered. MTX cessation resulted in complete resolution of the ulcers with no recurrences reported so far. The clinical and histopathologic features of MTX-induced oral ulcers are not always diagnostic and a detailed history and an extensive clinicopathologic investigation may be needed to exclude a lymphoproliferative disorder.

** Key words:**Atypical oral ulcers, ciprofloxacin, lymphoproliferative disorders, methotrexate.

## Introduction

Methotrexate (MTX), a well-known antimetabolite, functions as a folic acid antagonist and is widely used in high doses as a chemotherapeutic agent for the treatment of lymphomas, leukemias and some solid tumors (1). MTX is a well-established immuno-modulating drug that is administered in low doses (low-dose MTX-LDMTX), with concurrent folate supplementation for the treatment of chronic autoimmune and immune-mediated diseases such as rheumatoid arthritis, systemic lupus erythematosus and psoriasis (1,2).

LDMTX administration may be accompanied by a variety of adverse reactions. MTX toxicity is generally dose-dependent and rapidly dividing tissues, such as the bone marrow and the gastrointestinal tract are most commonly affected. Oral ulcerative stomatitis may be seen in about 14% of patients demonstrating a wide histopathologic spectrum that ranges from non-specific ulceration to lichenoid reactions to EBV (+/-) lymphoproliferative disorders (LPDs) (2,3).

A rare case of MTX-associated ulcerations is presented in a patient under long-term LDMTX that histopathologically exhibited atypical features mimicking a lymphoproliferative disorder.

## Case Report

A 73-year-old, non-smoker Caucasian female presented in the Department of Oral Medicine and Pathology complaining of pain-ful oral lesions of 5-day duration. The lesions had been developed after initiation of a *per os* ciprofloxacin course (500 mg 1x3) on 5th therapy day for an acute urinary tract *Escherichia coli* infection diagnosed 10 days ago. Two days before the patient’s visit in our clinic, the urologist recommended ciprofloxacin replacement by metronidazole and cefaclor. According to the patient, similar lesions had appeared in the past after ciprofloxacin intake, though she was not sure regarding the MTX intake at that particular time.

The patient had a known rheumatoid arthritis (RA) history diagnosed 15 years ago. Medication for the RA management consisted of a low-dose methotrexate therapy (2.5mg 1x1/week) supplemented with folic acid (5mg 1x1/week), prednisone 5mg 1x1/day, in addition to a 5-courses of rituximab IV (Mabthera inj.sol 500mg/50ml 1/week x2) administration; the last rituximab IV injection was given 10 months before the lesions presentation. Alendronic acid (5600iu/tab 1x1/weekly) and daily calcium supplementation were taken for osteoporosis management.

**Figure 1:**
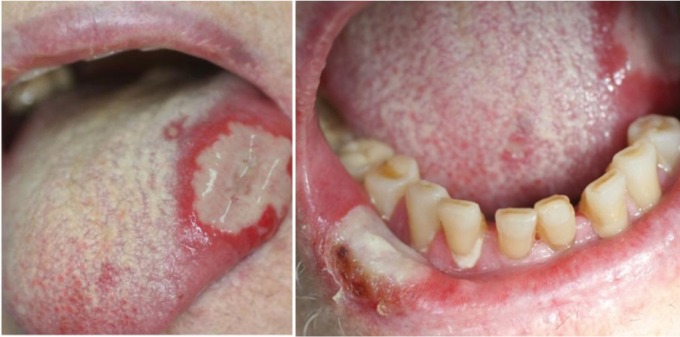
Atypical ulcers with irregular borders and yellow fibrinopurulent pseudomembrane on the dorsal surface of the tongue and the lower lip mucosa.

The oral examination revealed multiple ulcerative lesions of variable size and irregular shape, located on the dorsal and lateral borders of the tongue, the lower lip, the alveolar maxillary and mandibular mucosa, which were covered by a grey-whitish thick pseudo-membrane (Fig. 1), and showed a slightly vegetative ulcer base. There was no evidence of other mucocutaneous lesions or cervical lymphadenopathy. LDMTX-associated oral ulcers, drug-induced oral reaction, drug-induced erythema multiforme and a possible atypical viral or bacterial infection were considered in the differential diagnosis, taking into account the iatrogenic immunosuppression history, the abrupt onset of lesions, and the possible drug reaction (previous report of ciprofloxacin-induced oral lesions).

Tissue swabs from the ulcers were examined for viral, microbial and fungal infection, while a complete laboratory investigation was ordered. PCR tests for HSV, CMV and EBV were negative. A superimposed yeast infection by *geotrichum candidum* was detected, and interpreted to the long-standing iatrogenic immunosuppression. Complete blood test results were normal, including the folic acid levels. Elevated ESR and CRP levels and significant hypogammaglobulinemia (350 mg/dl, normal range: 700-1600 mg/dl) were noted. A lingual ulcer incisional biopsy was performed under local anesthesia.

**Figure 2:**
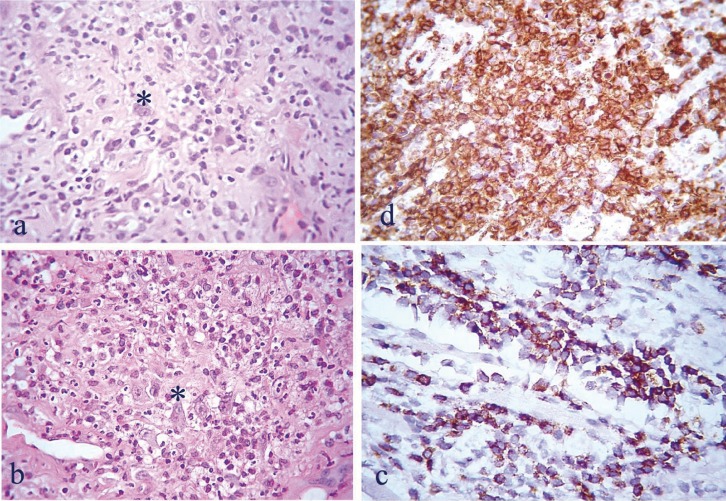
a) Diffuse mixed inflammatory infiltrate of the lamina propria by lymphocytes of varying size, histiocytes, neutrophils, and scarce eosinophils. b) Scattered irregularly-shaped binucleated atypical lymphocytes (Reed-Sternberg-like cells, asterisks) among the dense cellular infiltrate (H&E X250). Immunohistochemical evaluation revealed, among other markers, positivity for CD2 c) and CD4 d), (immunohistochemical stain X400).

Histopathologic examination revealed extensive ulceration of the covering stratified squamous epithelium extended deeply into subjacent striated muscle and adipose tissue that showed degeneration. The ulcer base was widely infiltrated by a dense polymorphous inflammatory cell population consisting of lymphocytes, histiocytes of variable size, along with neutrophils and scarce eosinophils (Fig. 2a). Among the inflammatory infiltration few scattered large binucleated lymphoid cells with noticeable atypical features were observed similar to Reed-Sternberg cells (Fig. 2b). Both the polymorphous inflammatory cells and the Re-ed-Sternberg-like cells exhibited positive immunostaining for T-cell markers CD2, CD3, CD4, CD8 and CD15 and negative for B-cell and NK-cell markers (CD20, CD30, Pax-5, CD56, TIA-1 and granzyme) (Figs. 2c-d). LMP-1 immunostaining and EBER test proved to be negative excluding the EBV presence. The above findings were suggestive of an EBV (-) lymphocytic infiltrate of T-cell phenotype. Based upon the patient’s medical history, the complete laboratory investigation, the clinical and histopathologic findings, a diagnosis of MTX-related non-specific ulcerative stomatitis was rendered, possibly induced by the recent ciprofloxacin administration.

**Figure 3:**
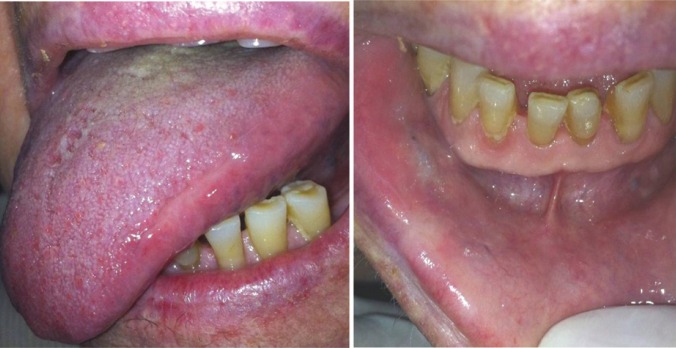
Resolution of the lingual and labial ulcers 3 weeks after MTX cessation.

With the rheumatologist’s consent, MTX intake was ceased and 3 weeks later the oral ulcers had healed completely (Fig. 3). Two months after MTX withdrawal, the drug was restarted, with no evidence of recurrence of the oral lesions noted so far.

The patient had provided written informed consent for publication of this case report and any accompanying images in a scientific journal, after the authors explained the possible benefits to dental science.

## Discussion

LDMTX adverse reactions may be present in 30-80% of the patients, while up to 30% discontinue the treatment as a consequence (2). Common side effects include myelosuppresion, nausea, diarrhea, abdominal pain, weight loss, and hepatotoxicity. The oral lesions seem to be dose-dependent, and a possible early sign of drug toxicity. Stomatitis has been referred in approximately 14%, and treatment discontinue in 3% of the patients, thus oral clinicians may be encountered with a MTX-induced lesion more often than previously thought (3). The risk of MTX-induced ulcers appears to be increased in patients with pre-existing folate deficiency. Elevated drug levels in the saliva acting topically are considered to promote the oral lesions development. Measurement of the excreted MTX concentration in the saliva has been proposed useful in predicting oral ulceration (3,4).

The LDMTX-associated oral ulcers may be apparent within the first few weeks, whereas in long-term toxicity the lesions can occur even years later in the disease course (2). MTX metabolism may be affected by age, compromised hepatic or renal function, whereas several contraindicated drugs may influence pharmacokinetics (1). Among them, quinolones and specifically ciprofloxacin may promote a reduced renal tubular MTX clearance resulting in MTX plasma levels elevation (5). In the current case, the ulcerative lesions appeared after a 5-day course of ciprofloxacin administration suggesting an acute MTX toxicity, despite the patient was under LDMTX treatment.

LDMTX-associated stomatitis may mimic various oral inflammatory conditions, infections or vesiculobullous diseases (3,6). In our case, the differential diagnosis included the ciprofloxacin-induced oral minor erythema multiforme (EM) due to patient’s history of similar oral lesions in the past, as well as herpes-associated EM, due to immunosuppresion. The possibility of oral EM seemed more unlikely based on the clinical features of the lesions, the lack of the EM characteristic hemorrhagic crusts on the lips vermillion border, and the gingival involvement that usually is not seen in EM.

The etiology of multiple, deep painful ulcers covered by thick necrotic pseudomembrane in medically immunocompromised patients may be associated with HSV, CMV or bacterial infection. Among the affected oral sites, dorsal tongue, hard palate, and/or gingiva have been referred as frequent locations (7). A possible infection was also considered in the differential diagnosis, but the microbiologic tests proved to be negative, additionally to the fact that the patient was already under a course of broad-spectrum antibiotics.

The interval between initiation of MTX therapy and MTX-induced LPDs varies, with most patients diagnosed after 3-5 years of continuous treatment (8). Several tissues and/or internal organs can be affected, including liver, kidneys, gastrointestinal mucosa, lung, spleen and skin. Besides the MTX-treatment associated neoplastic potential, the risk for hematopoietic malignancies, most often lymphoma, is 2-20-fold increased in patients with RA (3). Among them, MTX administration has been especially linked to the development of EBV-related LPDs (9).

The histopathologic diagnosis of LDMTX oral ulcers may be challenging, since the lesions comprise a wide spectrum of features (3,9). Twelve cases of oral LPDs in patients under LDMTX have been published in the English literature ( Table 1). Despite the well-established incidence of these lesions, to the best of our knowledge, this is the first case that was characterized by the finding of atypical Reed-Sternberg-like cells sharing features similar to a lymphoproliferative disorder, causing diagnostic dilemma. Atypical EBV negative cells simulating a lymphoma has not been referred in the previously published cases, though Kalantzis *et al.* described atypical cells in the ulcer base, but further specific analysis was not provided (3).

**Table 1:**
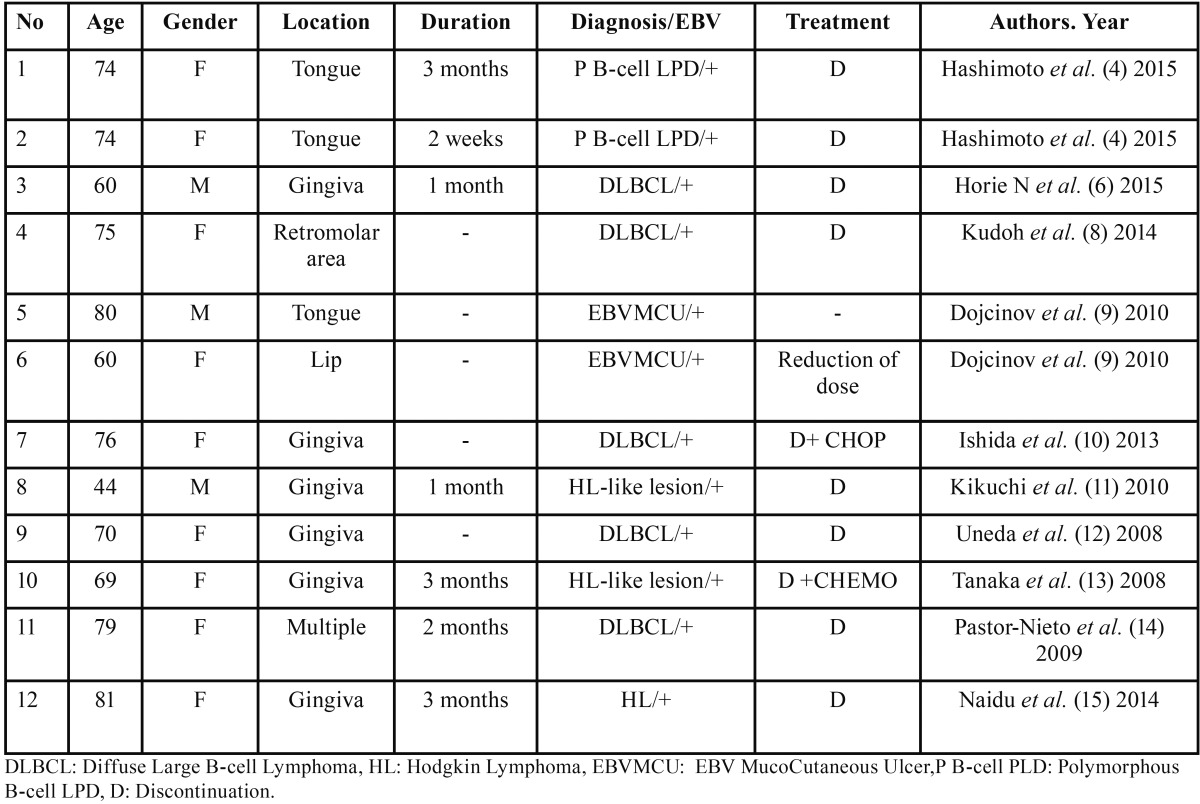
Reported cases of oral LDMTX-LPDs in the English-language literature.

Treatment of MTX-induced oral lesions consists of drug cessation or dose reduction, supplemented sometimes with folate administration, topical palliative therapies or conservative surgical excision. The gradual MTX polyglutamates clearance may result in healing of the ulcers within 2-3 weeks. A close follow-up course is of paramount importance, since recurrence of the lesions has been referred after a few months of drug removal in nearly 50% of patients (3,4,15).

In conclusion, a careful medical and pharmacologic history, along with a recent full blood test investigation is mandatory in patients under MTX intake. Clinicians should be aware of the possible side effects of LDMTX in the oral mucosa, as well as the drug pharmacokinetics, to avoid prescription of a contraindicated drug that may interfere with MTX metabolism.
